# Robot-assisted minimally invasive esophagectomy

**DOI:** 10.1007/s00104-016-0200-7

**Published:** 2016-07-28

**Authors:** R. van Hillegersberg, M. F. J. Seesing, H. J. F. Brenkman, J. P. Ruurda

**Affiliations:** Department of Surgical Oncology, University Medical Center Utrecht, Heidelberglaan 100, 3508 GA Utrecht, The Netherlands

**Keywords:** Esophageal cancer, Esophagectomy, Multimodal treatment, Surgery, robot-assisted, Thoracoscopy

## Abstract

Esophagolymphadenectomy is the cornerstone of multimodality treatment for resectable esophageal cancer. The preferred surgical approach is transthoracic, with a two-field lymph node dissection and gastric conduit reconstruction. A minimally invasive approach has been shown to reduce postoperative complications and increase quality of life. Robot-assisted minimally invasive esophagectomy (RAMIE) was developed to facilitate this complex thoracoscopic procedure. RAMIE has been shown to be safe with good oncologic results and reduced morbidity. The use of RAMIE opens new indications for curative surgery in patients with T4b tumors, high mediastinal tumors, and lymph node metastases after neoadjuvant treatment.

**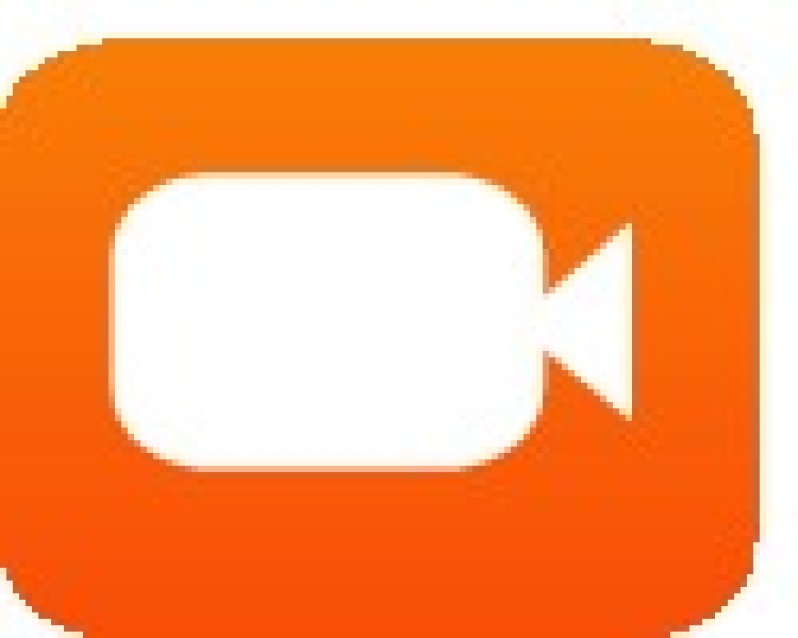
 **Video online**:**

The online version of this article (doi:10.1007/s00104-016-0200-7) contains a video, which is available to authorized users.

## Zusammenfassung

Die Ösophagolymphadenektomie ist der Eckpfeiler der multimodalen Behandlung des resektablen Ösophaguskarzinoms. Der chirurgische Zugang erfolgt bevorzugt transthorakal, mit 2-Feld-Lymphadenektomie und Rekonstruktion durch Magenhochzug. Für einen minimal-invasiven Ansatz wurde gezeigt, dass er die postoperativen Komplikationen reduziert und die Lebensqualität erhöht. Die roboterassistierte minimal-invasive Ösophagektomie (RAMIE) wurde mit dem Ziel entwickelt, dieses komplexe thorakoskopische Verfahren zu erleichtern. Die Sicherheit der RAMIE ist belegt, die onkologischen Ergebnisse sind gut, die Morbidität wird verringert. Die Anwendung eröffnet neue Indikationen für die kurative chirurgische Behandlung von Patienten mit T4b-Tumoren, Tumoren im oberen Mediastinum und Lymphknotenmetastasen nach neoadjuvanter Therapie.

## Background

Annually, 482,300 patients are diagnosed with esophageal cancer worldwide, and 406,800 patients die of this disease [1]. Esophagolymphadenectomy is the cornerstone of multimodality treatment for locally advanced esophageal cancer [2, 3]*.* The preferred curative treatment for esophageal cancer is transthoracic esophagectomy with a two-field lymph node dissection and gastric conduit reconstruction [4]. This procedure allows for en bloc resection of the esophagus and extensive mediastinal lymphadenectomy [5]*.*

Minimally invasive surgery was introduced to reduce surgical trauma and postoperative morbidity. Minimally invasive esophagectomy (MIE) has several advantages over open esophagectomy, such as diminished blood loss, reduced morbidity, and shorter hospital stay [6, 7]. However, conventional thoracolaparoscopic techniques are limited by a two-dimensional view, a disturbed eye–hand coordination, and a reduced freedom of movement. These technical limitations may compromise the feasibility of MIE and its worldwide adaptation [8–10].

In 2003, the robot-assisted thoracolaparoscopic approach was developed at the University Medical Center Utrecht (UMC Utrecht) in The Netherlands [10]*. *Robot-assisted minimally invasive esophagectomy (RAMIE) reduces the limitations of thoracoscopic esophagectomy by offering a stable three-dimensional (3D) view, 10-times-enlarged image, restored eye–hand axis, and excellent dexterity with the endowristed instruments. In this article we present our technique, give suggestions for successful implementation, discuss recent developments, and look to the future directions of RAMIE.

## RAMIE at UMC Utrecht

### Preparation

**Fig. 1:**
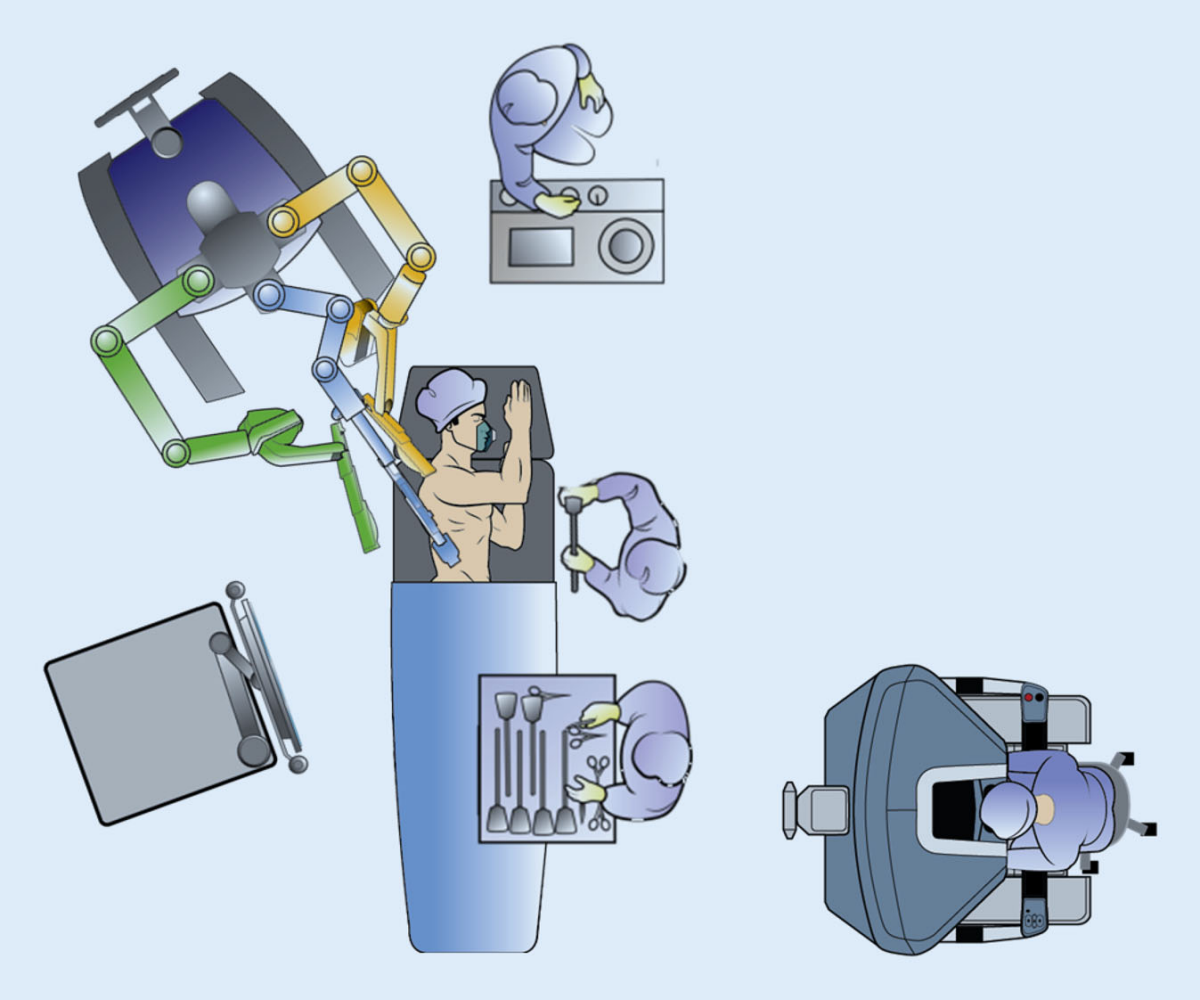
Operating room set-up. (© 2014 Intuitive Surgical Inc., used with permission)

**Fig. 2:**
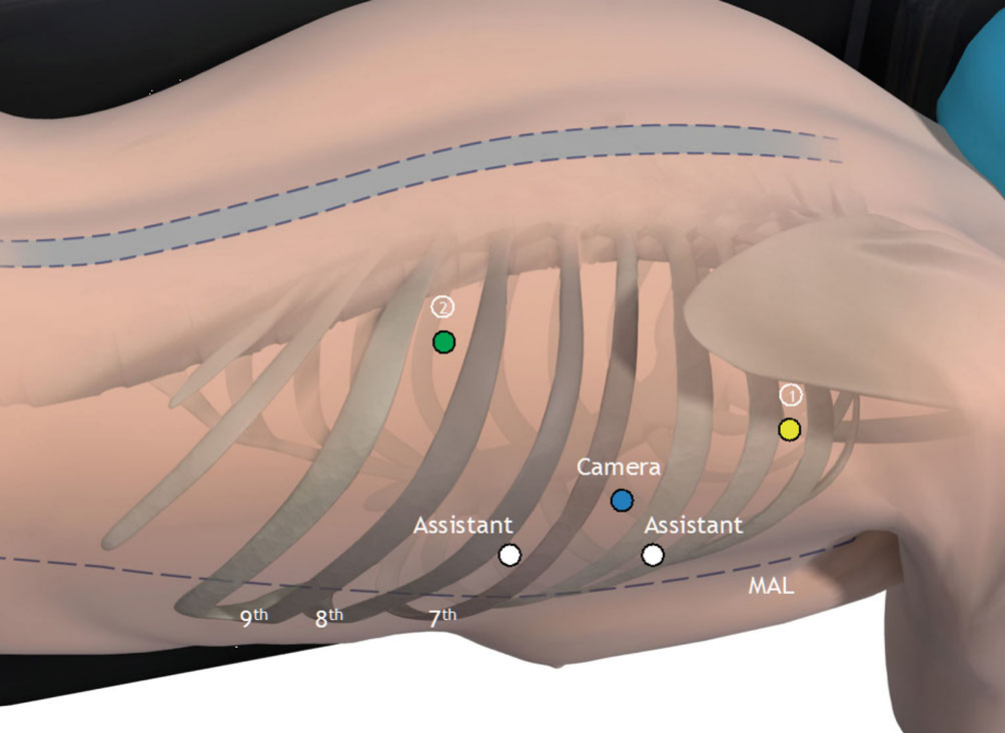
Trocar placement in the thoracic phase. Robotic arms *1* (*yellow*) and *2* (*green*), camera (*blue*), and two assisting ports (*white*). *MAL (midaxillary line)* (©2014 Intuitive Surgical Inc., used with permission)

General and thoracic epidural anesthetics are combined to ensure sufficient intraoperative and postoperative analgesia. Recently we started using single-dose and bilateral paravertebral block combined with sufentanil in the context of our enhanced recovery after esophagectomy program. This may provide similar postoperative analgesia and allow for early discharge avoiding the disadvantages of epidural anesthesia such as catheter malposition and hypotension [11]*. *The patient is intubated with a left-side double-lumen tube and is positioned in the left lateral decubitus position, tilted 45° to the prone position. The robotic system (da Vinci Si System, Intuitive Surgical Inc., Sunnyvale, Calif.) is positioned on the dorsocranial side of the patient (Fig. 1). Three ports are placed for the robotic system as well as two ports for the assisting surgeon (Fig. 2), whereafter the right lung is desufflated. Through one of the assistant ports, CO_2_ at 6 mmHg is insufflated to keep the lung out of the operative field.

### Surgical procedure

**Fig. 3:**
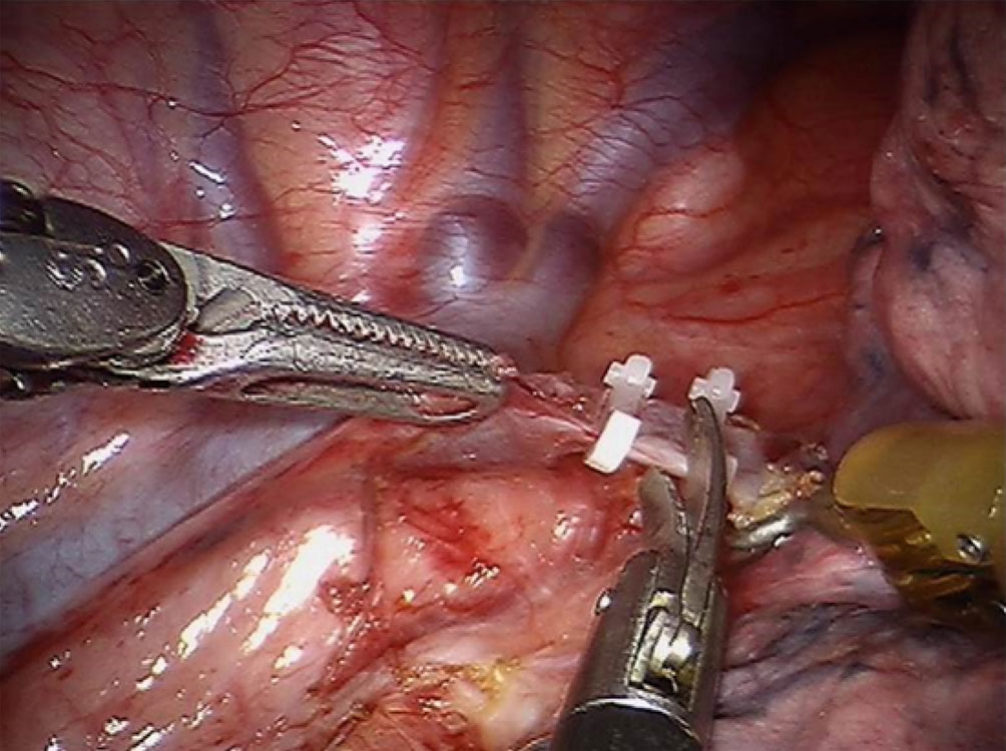
Clipping and dissection of the azygos vein. (From [30])

The right pulmonary ligament is divided and the parietal pleura is dissected at the anterior side of the esophagus from the diaphragm up to the level of the azygos arch. The azygos arch is ligated with robotic Hem-o-lok clips (Teleflex Medical, Durham, N.C.) and divided. These clips are endowristed facilitating precise positioning (Fig. 3). To establish dissection of the right paratracheal lymph nodes, dissection of the parietal pleura is continued above the azygos arch. The right vagal nerve is dissected below the level of the carina to preserve its pulmonary branches [12, 13]. Dissection of the parietal pleura is continued from cranially to caudally along the azygos vein at the posterior side of the esophagus. Paratracheally left, the left recurrent nerve is identified and carefully protected. At the level of the diaphragm, the thoracic duct is clipped with the robotic Hem-o-lok clips to prevent leakage. At this level, a Penrose drain is placed around the esophagus to facilitate traction and esophageal mobilization. The esophagus is then resected from the diaphragm up to the thoracic inlet en bloc with the surrounding mediastinal and subcarinal lymph nodes and the thoracic duct. The aortoesophageal arteries are identified and clipped.

**Fig. 4:**
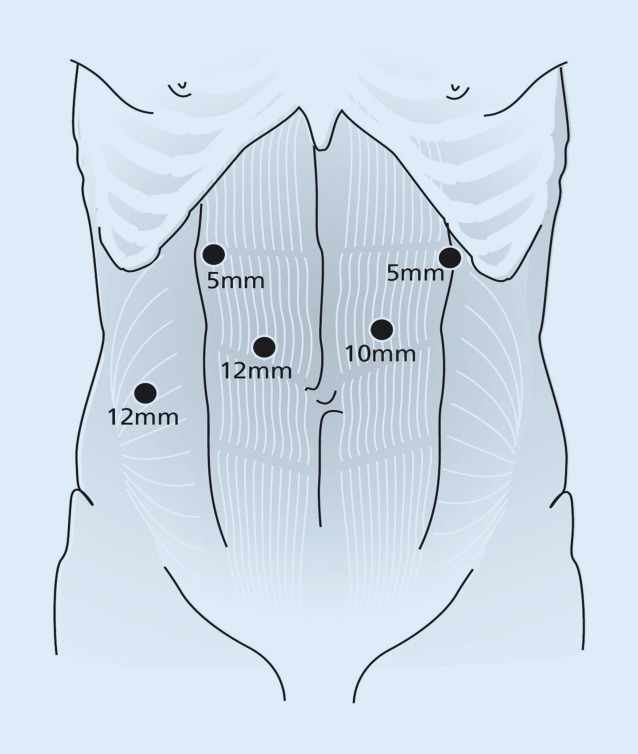
Trocar placement in the abdominal phase. (From [8], by kind permission of John Wiley and Sons)

The patient is put in supine position for the abdominal phase. After introduction of the five trocars (Fig. 4), the hepatogastric ligament is opened and transection of the lesser omentum is continued until the left crus of the diaphragm. Hereafter, the greater gastric curvature is dissected using a harmonic ace (Ethicon, Cincinnati, Ohio). Abdominal lymphadenectomy is performed including lymph nodes surrounding the celiac trunk, along the left gastric and splenic arteries and the lesser omental lymph nodes. The left gastric artery is ligated with Hem-o-lok and transected at its origin. Through a left-sided vertical incision along the sternocleidomastoid muscle, the cervical phase of the esophagectomy is initiated to facilitate mobilization of the cervical esophagus. No formal cervical lymph node dissection is carried out. The cervical esophagus is transected and a cord is attached to the specimen. The dissected esophagus with the surrounding lymph nodes en bloc are pulled down through the mediastinum under laparoscopic view. Hereafter, the left para-umbilical trocar port is widened to a 5–7-cm transverse incision. The resection specimen is removed through this incision with a wound drape (3M, St. Paul, Minn.) to create the gastric conduit extracorporeally. A linear stapler (GIATM 80, 3.8 mm; Medtronic, Minneapolis, Minn.) is used to create a 4-cm-wide gastric conduit, which is routinely oversewn with a 3/0 polydioxanone single-layer running suture. The gastric conduit is pulled up through the mediastinum along the original anatomic tract of the esophagus with the aid of a plastic tube (laparoscopic camera bag). A cervical hand-sewn end-to-side anastomosis is created between the gastric tube and the cervical esophagus using a 3/0 polydioxanone single-layer running suture. A jejunostomy feeding tube is placed in the second loop after the ligament of Treitz for postoperative feeding. The abdomen is closed in layers with a PDS loop for the fascia and skin intracutaneously with Monocryl.

## Pushing the limits of esophagectomy with RAMIE

Since the introduction of RAMIE, we have gained considerable experience with the use of the da Vinci robot in over 300 cases. After a learning curve of 120 cases, a plateau was reached with a steady level of performance. Utilizing the full capabilities of the da Vinci robot, we are currently investigating new indications in patients who were deemed inoperable with conventional surgery.

**Fig. 5:**
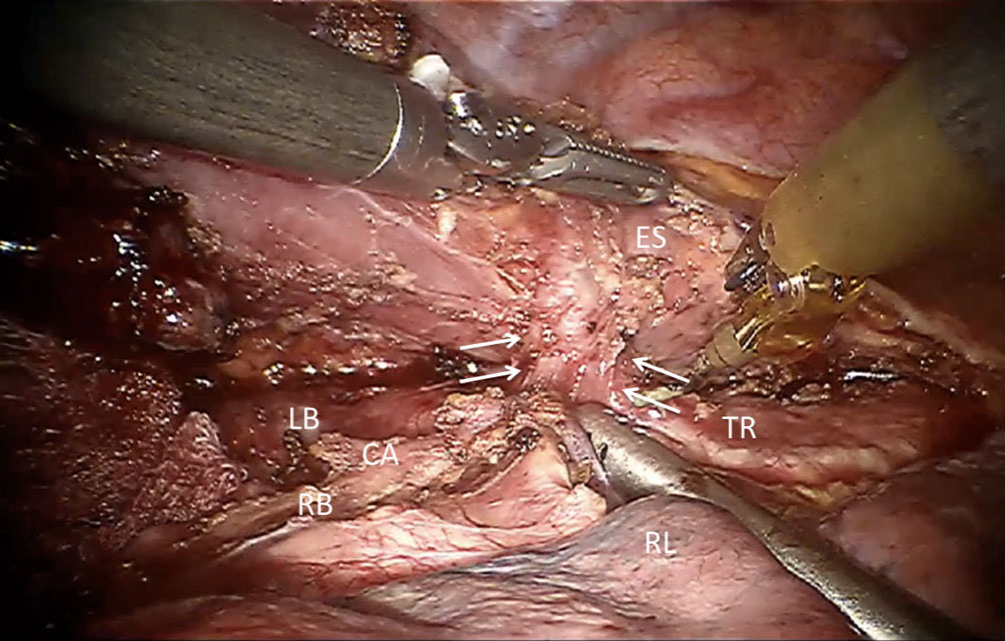
T4b esophageal tumors, tracheal involvement. *ES* esophagus, *CA* carina, *LB* left bronchus, *RB* right bronchus *TR* trachea, *RL* right lung

Until recently, patients with cT4b tumors were considered inoperable and guidelines recommend definitive chemoradiotherapy as the treatment of choice [14]. However, cT4 status is a negative predictor of locoregional control [15]. Definitive chemoradiotherapy is associated with a high rate of esophageal stenosis and perforation, the latter occurring in 25 % of patients, and most often these perforations are lethal. Furthermore, functional results are poor and there is frequently recurrence in up to 41 % of cases [15, 16]. Therefore, we started salvage surgery with RAMIE in patients with cT4b esophageal tumors after long-course chemoradiotherapy. The patients are restaged with positron emission tomography–computed tomography and endobronchial ultrasound to determine the actual situation, and they are selected for salvage surgery if tracheal ingrowth has been reduced (Fig. 5). The enlarged 3D image allows for very precise dissection of the irradiated tumor tissue from the trachea, bronchi, and aorta. The level of precision makes dissection in downstaged T4b tumors feasible. So far, we have treated ten patients using this strategy, leading to radical resections in 90 % of cases (unpublished data). We are awaiting the long-term oncologic and functional results with this approach before it can be recommended for all patients.

**Fig. 6:**
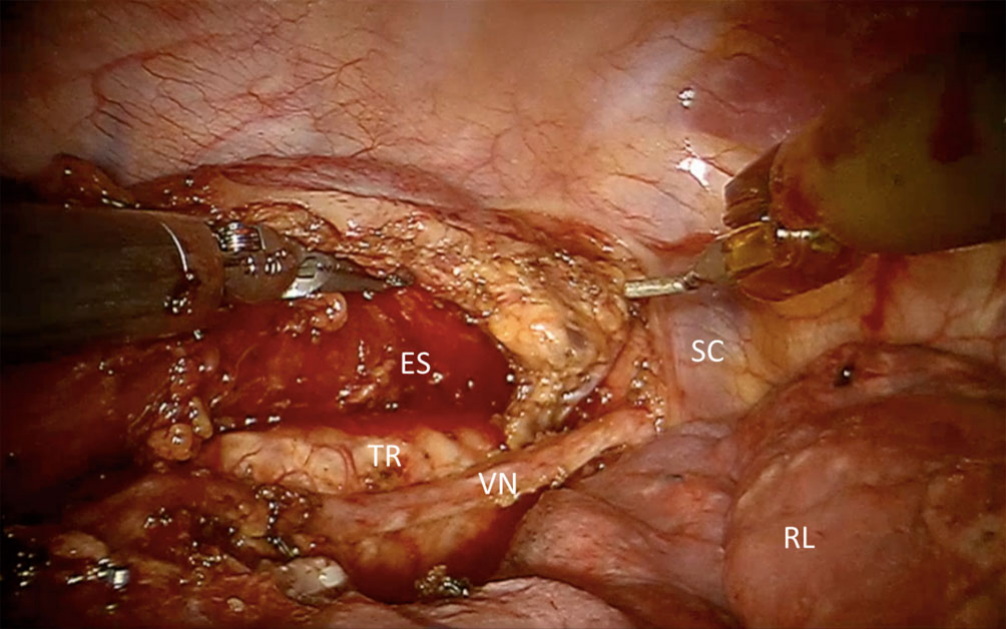
Upper mediastinum.* ES* esophagus, *VN* vagal nerve, *TR* trachea, *RL* right lung, *SC* subclavian artery

Moreover, the upper mediastinum and thoracic aperture can be reached with an excellent 3D view and magnified observation of the operative field (Fig. 6; [17]). In this way, we were able to achieve an R0 resection in 28 out of 29 patients (97 %) with upper esophageal tumors and paratracheal lymph node involvement (unpublished data).

Future directions include the use of the robotic platform for image-guided surgery and fluorescence detection of lymph nodes and tumor margins. The integration of optical imaging modalities that specifically visualize the areas of interest may reduce complications and improve the surgical accuracy of lymph node dissection [18].

Another application of intravascular fluorescence dye imaging is the intraoperative assessment of the vascularization of the gastric conduit. This may guide one to the optimal site of the anastomosis and thereby contribute to reducing anastomotic leakage [19].

## Surgical training program

We have developed a structured training program that enables esophageal surgeons to be guided through the learning curve in 20 cases*. *The proctored surgeons should have basic minimally invasive skills and knowledge of esophageal surgery. The program starts with three case observations in a RAMIE expert center, followed by a basic robotic course and a cadaveric laboratory course. Thereafter, the proctor supervises the surgeon for the first three to ten cases and reviews his skills after the first 20.

Optimal performance during technically complex procedures requires a dedicated team [20]. Therefore, at least two motivated surgeons, a dedicated anesthetist, and a scrub nurse should be involved in the program. A sufficient case load (>20/year) and guaranteed access to a robotic system are of crucial importance [21, 22].

In urology, proctoring has proven to be an essential mechanism for the successful implementation of robot-assisted radical prostatectomy, and this approach is comparable in RAMIE [23].

## Discussion

RAMIE offers a 3D, magnified surgical view combined with a high degree of freedom of the articulating instruments. This facilitates meticulous dissection from the diaphragm to the thoracic inlet. Especially for the thoracic phase, where the esophagus is surrounded by delicate vital structures such as the trachea, pulmonary veins, aorta, and recurrent nerves, these features are of great value. Moreover, these features allow the exploration of salvage surgery in patients with previously inoperable T4b and upper mediastinal tumors.

Robotic assistance may also facilitate the use of an intrathoracic anastomosis [24]. Until recently, we performed a three-stage esophagectomy (McKeown procedure) with a hand-sewn esophagogastric anastomosis in the left side of the neck as stated previously. However, the incidence of anastomotic leakage after cervical esophagogastrostomy remains relatively high (10–30 %) and intrathoracic manifestation occurs in more than half of patients with cervical anastomotic leakage [25, 26]. The incidence of leakage from intrathoracic anastomosis is reported to be lower [27]. Therefore, we started performing a two-stage (Ivor Lewis) procedure with a robotic hand-sewn intrathoracic anastomosis for distal esophageal tumors. Constructing an intrathoracic anastomosis during conventional thoracoscopy is technically challenging. By working in the upper thoracic aperture, the instruments have to reach deep into the thorax, imposing problems in manipulation through the fulcrum effect at the ribs [28]. The robot nullifies these problems owing to the endo-wristed intracorporeal instruments, tremor filtering, and its 3D view of the surgical field. Therefore, in our opinion the robot contributes to a high-quality intrathoracic anastomosis. In our initial experience, we have found the hand-sewn robotic technique to be very controlled and reliable. Larger numbers of patients have to be treated with this technique before the success rates and long-term results can be reported.

So far, we have chosen to perform the abdominal aspect of our procedure via a laparoscopic approach, since the da Vinci Si Surgical System (Intuitive Surgical Inc.) was not constructed to facilitate multiquadrant surgery. The new da Vinci Xi Surgical System (Intuitive Surgical Inc.) has its four arms mounted on an overhead boom enabling multiquadrant surgery without the need to redock the system. Results of the robotic abdominal phase are awaited in the near future.

## Conclusion

In conclusion, robotic-assisted minimally invasive esophagectomy with two-field lymphadenectomy is an excellent minimally invasive technique for dissecting the esophagus from the mediastinum with radical lymphadenectomy. Additionally, it may provide new curative options in patients deemed inoperable with conventional surgery. Robotic assistance reduces the limitations of MIE while retaining its advantages over open esophagectomy. Therefore, we initiated a randomized controlled trial, comparing robot-assisted and open three-phase esophagectomy to measure the additional value of RAMIE over open esophagectomy [29]. Short-term results are expected in the course of 2016.
